# Adolescent Δ^9^-THC Exposure Differentially Affects Mice Depending on Their Personality

**DOI:** 10.3390/ph19071009

**Published:** 2026-06-29

**Authors:** Dilorom Begmatova, Liudmila Vinnikova, Natalya Zemliana, Kenneth Blum, Panayotis K. Thanos, Natalya M. Kogan, Albert Pinhasov

**Affiliations:** 1 Department of Molecular Biology, Adelson School of Medicine, Ariel University, Ariel 4070000, Israel; dilorom0508@gmail.com (D.B.); liudmilavi@ariel.ac.il (L.V.); nataliaz@ariel.ac.il (N.Z.); drd2gene@gmail.com (K.B.); 2 Behavioral Neuropharmacology and Neuroimaging Laboratory on Addictions, Clinical and Research Institute on Addictions, Department of Pharmacology and Toxicology, Jacobs School of Medicine and Biosciences, University at Buffalo, Buffalo, NY 14203, USA; thanos@buffalo.edu; 3 Department of Psychology, University at Buffalo, Buffalo, NY 14260, USA

**Keywords:** dominant-submissive relationship, cannabis, THC, adolescence

## Abstract

**Background:** Adolescence is a sensitive period for brain maturation during which exposure to Δ^9^-tetrahydrocannabinol (THC) can induce long-lasting neurobehavioral alterations. Yet, preclinical and clinical studies report inconsistent long-term outcomes of adolescent THC exposure, ranging from clear impairments to apparently normalizing effects. We hypothesize that these discrepancies reflect stable individual differences in stress-coping abilities. **Methods:** To test this, selectively bred Dominant (Dom; stress-resilient, risk-prone) and Submissive (Sub; stress-vulnerable, depressive-like) Sabra mice received THC or vehicle during adolescence and were assessed in adulthood. **Results:** Anxiety-like and exploratory behavior, measured in the hole-board test, were differentially affected by THC as a function of stress vulnerability: in Sub mice, THC increased exploration and reduced anxiety-like behavior, whereas in Dom mice THC produced the opposite pattern. Recognition memory, evaluated by the novel object recognition test, showed modest, line-dependent alterations. Sensitivity to N-methyl-D-aspartate (NMDA) receptor hypofunction, a widely used index of vulnerability to schizophrenia-like symptoms, was examined using MK-801-induced locomotion. Adolescent THC potentiated MK-801-evoked hyperlocomotion in Dom mice but attenuated it in Sub mice. **Conclusions:** In the context of increasing medical and recreational cannabis exposure, these in vivo findings suggest that stress-vulnerability-related traits may be an important variable to consider in future preclinical and translational studies of adolescent THC exposure.

## 1. Introduction

Stress and inflammation are important contributors to the development of neuropsychiatric disorders. A growing body of evidence indicates that early life stress, whether prenatal or postnatal, can alter brain function and increase the risk of anxiety, depression, schizophrenia, and other psychiatric conditions later in life [1,2]. Stress activates the hypothalamic–pituitary–adrenal (HPA) axis, leading to the release of glucocorticoids such as cortisol, which influence multiple neurodevelopmental processes [3,4]. Prolonged exposure to stress and the associated inflammatory responses may induce neuroinflammatory cascades, oxidative stress, and excitotoxicity, further impairing brain function [5,6,7,8,9,10].

The relationship between stress and psychiatric disease is not uniform across individuals. Instead, individuals vary in their resilience or vulnerability to stress-induced pathologies. Resilience refers to the ability to maintain or regain mental health despite adversity, while vulnerability reflects a predisposition to develop psychiatric symptoms following exposure to stressors [11,12]. In rodent models, resilience and vulnerability have been linked to differences in neuroendocrine function, neuroimmune responses, neurotransmitter systems, and behavior under both basal and stress conditions [13].

Postnatal brain development is a complex and dynamic process that continues into early adulthood [14]. During this period, the brain undergoes significant structural and functional changes, including synaptic pruning, myelination, and maturation of neurotransmitter systems [15]. These changes make the adolescent brain particularly sensitive to environmental influences. Exposure to environmental toxins such as cannabinoids during adolescence may disrupt neurodevelopment, interfere with neural circuity, and contribute to cognitive and behavioral impairments [16,17,18,19]. Prospective, longitudinal, epidemiological studies suggest that adolescent cannabis use increases the risk for psychosis and other neuropsychiatric disorders [17], particularly when use begins before the age of 16 years [20]. This period of heightened sensitivity coincides with the developmental maturation of the endocannabinoid system, which plays a fundamental role in brain development [21]. The main psychoactive component of cannabis is Δ^9^-tetrahydrocannabinol (THC), which activates the endocannabinoid system primarily via CB1 (and to some extent CB2) receptors [22,23]. Activation of CB1 receptors is thought to create an imbalance in excitatory–inhibitory signaling in the brain by influencing the GABAergic, glutamatergic, and dopaminergic neurotransmission [24]. Even if cannabis exposure in adolescence increases risk of psychosis, only a minority of cannabis users develop the disorder, suggesting that cannabis may interact with pre-existing inherited vulnerabilities and increase the risk for neuropsychiatric illness [25].

Among the neuropsychiatric outcomes potentially linked to adolescent cannabis exposure, schizophrenia is one of the most studied. Schizophrenia has been increasingly associated with glutamatergic dysfunction, particularly hypofunction of NMDA receptors [26,27]. A commonly used pharmacological model of NMDA receptor hypofunction is MK-801 (also known as dizocilpine), a non-competitive NMDA receptor antagonist. In animal models, administration of MK-801 induces hyperlocomotion and stereotypic behaviors, serving as a widely accepted proxy for schizophrenia-like phenotypes. Notably, adolescent exposure to THC has been shown to enhance sensitivity to MK-801-induced hyperactivity in some rodent strains, supporting the hypothesis that cannabinoids may exacerbate or unmask underlying glutamatergic vulnerabilities [28,29]. This aligns with clinical data indicating that cannabis use during adolescence can increase the risk of developing schizophrenia, particularly in individuals with a genetic or neurodevelopmental predisposition [25].

In this study, we used a unique mouse model characterized by social dominance and social submissiveness that represent resilience and vulnerability to stress respectively. This model was developed through selective breeding and social interaction paradigms. Dom and Sub mice exhibit differential response to environmental stressors and psychotropic agents, age-dependent differences in cognitive abilities and distinct forms of synaptic plasticity, including short- and long-term potentiation [30,31]. Dom and Sub mice have proven to be a valuable tool to study personality-based responses to psychotropic agents [31,32]. Here, we aimed to explore how dominance and submissiveness might influence the response to sub-chronic THC administration during adolescence.

## 2. Results

### 2.1. Adolescent THC Attenuates Body Weight Gain in Dom and Sub Mice

**Figure 1:**
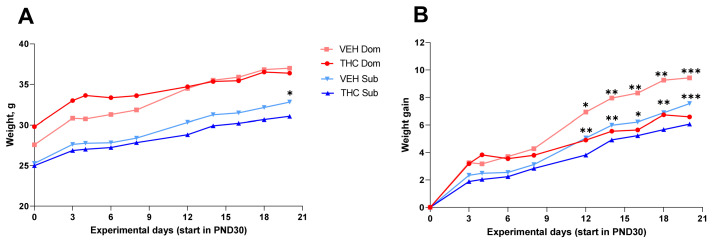
Adolescent administration of THC decreased body weight gain of Dom and Sub mice. (**A**) Body weight. (**B**) Weight gain. Statistical significance between groups was assessed using two-way ANOVA with treatment effects within strain analyzed by Sidak test. Error bars indicate SEM (*n* = 10 per group). * *p* < 0.05; ** *p* < 0.01; *** *p* < 0.001.

Prolonged adolescent exposure to THC (8 mg/kg, s.c., daily for 21 days) significantly reduced body weight gain in both Dom and Sub mice, as illustrated in Figure 1 (Figure 1A, body weight trajectory, Dom: F_9,180_ = 15.59; *p* < 0.0001, S: F_1,180_ = 5.685; *p* = 0.0181; Sub: F_9,180_ = 63.45; *p* < 0.0001, S: F_1,180_ = 34.29; *p* < 0.0001; day 21 *p* = 0.0179; Figure 1B, rate of body weight gain, Dom: F_9,180_ = 54.52; *p* < 0.0001, S: F_1,180_ = 32.61; *p* < 0.0001, I: F_9,180_ = 3.814; *p* = 0.0002; Sub: F_9,180_ = 197.1; *p* < 0.0001, S: F_1,180_ = 55.94; *p* < 0.0001, I: F_9,180_ = 2.567; *p* = 0.0084). Significant reduction in weight gain was observed after 2 weeks of THC treatment until the last day of injection in both Dom and Sub mice.

### 2.2. Adolescent THC Increased Sub Mouse Exploratory Activity

**Figure 2:**
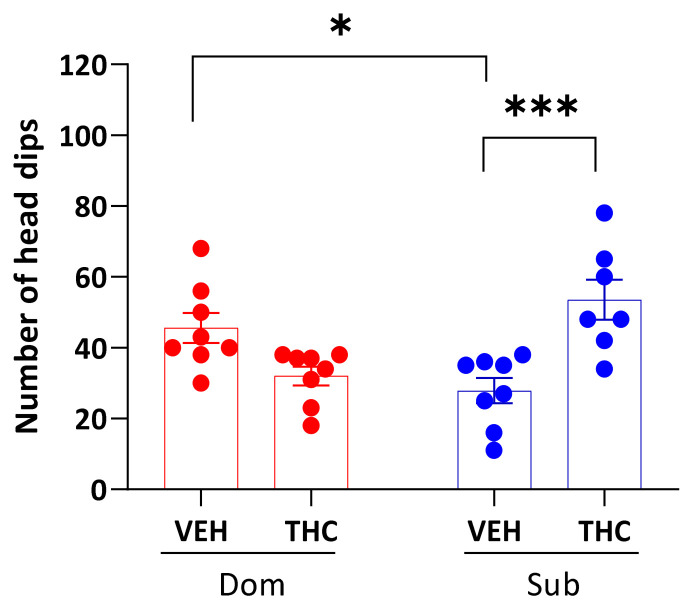
Adolescent THC increased Sub mice exploratory activity in HBT. Statistical significance between groups was assessed using two-way ANOVA with Sidak test. Error bars indicate SEM (*n* = 7–10 per group). * *p* < 0.05; *** *p* < 0.001.

Adolescent THC administration showed a significant increase in head-dipping behavior in Sub mice compared with corresponding saline-treated group in the hole-board test (HBT). In contrast, no effect on head-dipping behavior was observed in Dom mice. Sub control mice exhibited less exploratory activity compared with corresponding Dom groups. (Figure 2, I: F_1,27_ = 23.32, *p* < 0.0001, Dom: *p* = 0.0243, Sub *p* = 0.0009). No effect of THC was detected in EPM test (Supplementary Figure S1).

### 2.3. Adolescent THC Increased Short-Term Memory in Sub Mice

**Figure 3:**
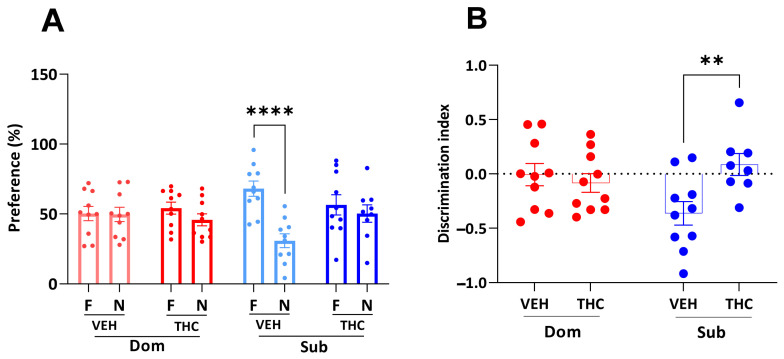
Adolescent THC increased short-term memory in Sub mice in NORT. (**A**) Preference (%) to the object and (**B**) discrimination index (DI). Statistical significance between groups was assessed using two-way ANOVA with Sidak test. Error bars indicate SEM (*n* = 7–10 per group). ** *p* < 0.01; **** *p* < 0.0001.

A significant effect of adolescent THC treatment was observed during performance of the short version of the novel object recognition test (NORT). Thus, 30 min after exposure to two identical objects, Sub control group spent significantly less time near the novel object compared to the familiar object, while no significant differences between time spent near the familiar or novel object was observed among other groups (Figure 3A, I: F_3,70_ = 4.946, *p* = 0.0036; [THC effect] F_1,70_ = 9.682, *p* = 0.0027; Sub: *p* < 0.0001). Sub THC group exhibited increased discrimination index compared to their respective control, with no changes observed in Dom groups. (Figure 3B, I: F_1,34_ = 6.862, *p* = 0.0131; Sub: *p* = 0.0082).

### 2.4. Adolescent Sociability Decreased in Dom and Increased in Sub Mice Exposed to THC

**Figure 4:**
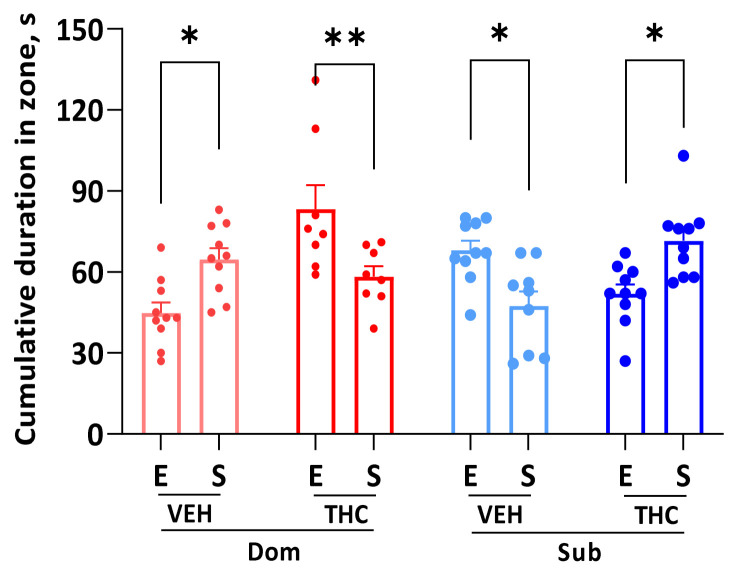
Adolescent THC decreased sociability in Dom and increased in Sub mice in TChT. Statistical significance between groups was assessed using two-way ANOVA with Sidak test. Error bars indicate SEM (*n* = 7–10 per group). * *p* < 0.05; ** *p* < 0.01. Abbreviations: E—empty room, S—room with stranger.

Effect of THC on social behavior was tested using the three-chamber test (TChT). Exposure to THC in Dom mice significantly decreased social preference compared to the vehicle group (Figure 4). In contrast, Sub mice significantly increased dwell time with stranger after adolescent exposure to THC compared to their vehicle-treated group. Social behavior in rodents is a key measure for modeling neuropsychiatric and affective disorders [33,34]. No significant effect of adolescence THC exposure was detected in Y-maze and FST (Supplementary Figures S2 and S3).

### 2.5. Adolescent THC Decreases MK-801-Induced Locomotor Activity in Sub Mice

**Figure 5:**
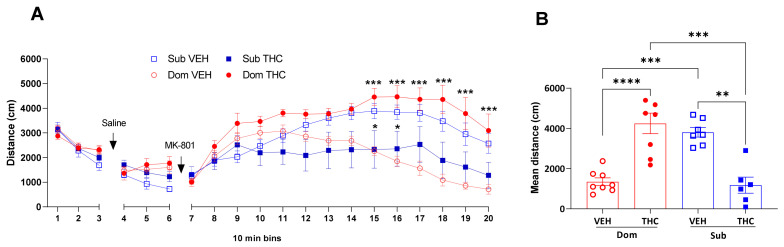
Adolescent THC decreases MK-801-induced locomotor activity in Sub mice. (**A**) Distance traveled over time following THC treatment. (**B**) Mean distance traveled (150–180 min window). Statistical significance between groups was assessed using two-way ANOVA with treatment effects within strains (**A**) or across all groups (**B**) analyzed by Sidak test. Error bars indicate SEM (*n* = 7–10 per group). * *p* < 0.05, ** *p* < 0.01; *** *p* < 0.001; **** *p* < 0.0001.

The effects of adolescent THC treatment on the locomotor response to MK-801 (0.5 mg/kg) were determined. As demonstrated in Figure 5, Adolescent THC significantly altered locomotor activity, which increased in Dom mice and decreased in Sub mice.

## 3. Discussion

The present study demonstrates that prolonged adolescent exposure to THC produces divergent long-term behavioral outcomes depending on inherited stress-coping traits, as modeled by Dominant (Dom) and Submissive (Sub) mice [30,31]. These findings provide a potential explanation for the heterogeneity reported in both preclinical and clinical studies of adolescent cannabis exposure, suggesting that stable personality-like traits critically shape the direction and magnitude of THC-induced effects. Cannabis is the world’s most widely used illicit drug with increasing consumption following its medical and recreational legalization [35,36,37,38]. Accumulating evidence indicates that THC is a potent psychotropic agent capable of inducing lasting neurobehavioral alterations, particularly when exposure occurs during adolescence, a critical window of brain maturation [39,40,41], the diversity and severity of which strongly vary among the genetically heterogeneous human population [42,43]. Moreover, adolescence is a peak period for first-time drug use, predominantly for cannabis, given the perception of the drugs’ ‘harmless’ side-effects and ease of access [44]. Importantly, the variability in outcomes observed across individuals suggests that cannabis-related risks cannot be fully understood without considering inter-individual differences in vulnerability and resilience. In this context, our data show that adolescent THC exposure significantly reduced body weight gain in both Dom and Sub mice, consistent with recent reports describing metabolic alterations in adulthood following adolescent exposure to low-dose THC [45,46]. While this “pseudo-lean” state may superficially resemble a beneficial metabolic state, it likely might be rooted in dysregulation of energy balance and adipose organ dysfunction [19,45,46]. Although weight reduction was similar across lines, behavioral responses diverged markedly. Thus, a central finding of this study is the bidirectional effect of THC on social and exploratory behaviors. Specifically, THC significantly increased sociability and exploratory activity in Sub mice, whereas it reduced sociability in Dom mice. This pattern suggests that THC may exert anxiolytic-like effects in stress-vulnerable individuals, while inducing adverse or dysregulating effects in stress-resilient, risk-prone individuals. The absence of significant THC effects in the EPM, Y-maze, and FST should also be considered when interpreting the behavioral profile. These negative findings indicate that adolescent THC did not produce a generalized alteration across all anxiety-like, cognitive, or stress-coping assays. Rather, the effects were domain- and assay-specific, emerging most clearly in the HBT, NORT, TChT, and MK-801-induced locomotor response. This pattern is not necessarily contradictory, because these tests differ in behavioral demands, stress load, motivational components, and neural substrates. For example, the HBT measures novelty-driven exploratory head-dipping behavior, whereas the EPM depends more strongly on conflict between open-space avoidance and exploration. Similarly, the NORT assesses object-recognition memory, whereas spontaneous alternation in the Y-maze reflects spatial working memory and exploratory sequencing. Finally, the FST measures acute stress-coping strategy and may be less sensitive to the specific long-term THC-induced changes detected in social behavior, object recognition, and pharmacological sensitivity to NMDA receptor blockade. Thus, the negative tests help define the specificity of the THC phenotype rather than weakening the overall conclusion. Human studies report dose- and individual-dependent effects of THC, ranging from relaxation to anxiety and psychosis-like symptoms. Thus, it reduced social anxiety when volunteers were exposed to fearful and angry faces at low doses [47], yet at higher doses (>10 mg) THC the effect was opposite [48]. One possible explanation is that THC may differentially influence limbic, paralimbic, mesolimbic, and glutamatergic systems in Dom and Sub mice [49,50]; however, this interpretation remains hypothetical because these mechanisms were not directly examined in the present study. Our findings extend this framework by demonstrating that baseline stress-coping phenotype determines the functional outcome of cannabinoid exposure. These behavioral differences are further supported by cognitive and pharmacological findings. It was recently shown by our group that behavioral outcomes of short-term THC exposure are different in Dom and Sub mice. Thus, Sub mice demonstrated a significant place-aversion in a conditioned place preference paradigm, whereas Dom displayed no behavioral changes [51]. However, at lower doses THC was able to reduce depressive-like behavior in Sub mice [51]. These findings are in good agreement with human studies showing that THC affects individuals differently: some individuals experience relaxation, whereas others develop psychotic states [52,53]. As stated above, the behavioral differences to psychotropic drugs in Dom and Sub mice likely reside jointly in their stress-response patterns and mesolimbic system function. For example, a differential effect between Dom and Sub mice was also observed in response to cocaine under naïve and stressful conditions [54]. These behavioral differences are further supported by cognitive and pharmacological findings. THC improved short-term recognition memory in Sub mice but not in Dom mice, suggesting a selective normalization of cognitive function in stress-vulnerable individuals [51]. In contrast, Dom mice exhibited increased sensitivity to NMDA receptor hypofunction, as reflected by enhanced MK-801-induced hyperlocomotion, whereas Sub mice showed an attenuated response [55]. Given that NMDA receptor dysfunction is a well-established model of schizophrenia-related pathology, these results imply that adolescent THC exposure may exacerbate glutamatergic vulnerability in resilient phenotypes while potentially buffering it in vulnerable ones. The mechanistic interpretation of these findings remains hypothetical. Although the divergent behavioral responses observed here may involve phenotype-dependent modulation of endocannabinoid, glutamatergic, dopaminergic, or stress-response pathways, the present study did not directly assess receptor expression, neurotransmitter release, synaptic plasticity, neuroinflammation, HPA-axis activity, or circuit-level changes. Taken together, our findings suggest that the long-term impact of adolescent THC exposure cannot be understood solely in terms of dose, age at exposure, or generic vulnerability, but must also be considered in relation to stable inter-individual differences in stress coping and social dominance. Submissive, stress-vulnerable mice exhibited a pattern of metabolic and behavioral changes that in some domains may be interpreted as partial normalization, whereas dominant, stress-resilient mice showed evidence of THC-induced disruption of affective and cognitive function. The data suggests that stable stress-coping traits can modify the direction of long-term THC-associated behavioral outcomes in this mouse model. Whether analogous trait-dependent effects occur in humans requires dedicated clinical and longitudinal studies. While certain individuals may experience transient or domain-specific benefits, others may be at increased risk for long-term neuropsychiatric disturbances, particularly when exposure occurs during adolescence [23,25,56]. Nevertheless, these findings should be interpreted with caution and several limitations of the Dom/Sub model should be acknowledged. Although selectively bred Dominant and Submissive mice provide a useful experimental framework for studying stable differences in stress-coping style, social rank, and vulnerability/resilience-related behavioral traits, these phenotypes should not be considered direct equivalents of human personality dimensions or psychiatric categories. Human temperament, stress vulnerability, and cannabis responsiveness are shaped by complex genetic, developmental, environmental, and sociocultural factors that cannot be fully reproduced in a selectively bred mouse model. Moreover, the dose and regimen of THC used in this study may not fully replicate patterns of human cannabis use. Therefore, the Dom/Sub model should be viewed as a reductionist preclinical tool for testing trait-dependent cannabinoid sensitivity rather than as a direct translational model of human cannabis responses. Future studies should aim to integrate genetic, environmental, and developmental factors to further refine our understanding of individualized responses to cannabinoid exposure.

In conclusion, this study demonstrates that personality-like traits fundamentally shape the long-term consequences of adolescent THC exposure, influencing behavioral and cognitive outcomes in mice. These findings underscore the need for temperament-informed risk assessment and caution in adolescent cannabis use, particularly in the context of increasing societal acceptance and medicalization of cannabis.

## 4. Materials and Methods

### 4.1. Animals

30-days-old Dominant (Dom) and Submissive (Sub) (selectively bred over 53 generations) male mice [30,31] used in this study. Animals were given standard laboratory chow and water ad libitum in a colony room maintained on a 12:12 L:D cycle (lights on 07:00–19:00 h). The experimental unit was an individual mouse. Animals were housed in groups of five per cage, with each experimental group consisting of 10 mice (two cages per group). Sample sizes were selected based on previous studies using the same animal model and experimental procedures, which demonstrated that this number of animals was sufficient to detect biologically relevant differences between groups. A total of 160 mice were used in the study. No a priori inclusion or exclusion criteria were established. All animals that completed the experimental procedures were included in the analyses. The exact sample size for each analysis is reported in the corresponding figure legends and results section. Animals were randomly allocated to experimental groups. Randomization was not applied, as animals were assigned to groups according to phenotype and treatment conditions. Potential confounding factors were minimized by maintaining identical housing conditions, testing animals during the same period of the light cycle, and applying standardized experimental procedures to all groups. Blinding was not performed. The investigator was aware of group allocation throughout the experiment, including treatment administration, outcome assessment, and data analysis. The study was exploratory in nature; therefore, no primary outcome measure was predefined, and no formal sample size calculation was performed.

The present study received approval from the Ariel University Institutional Animal Care and Use Committee (protocol numbers: IL-178-03-19; IL-177-02-19).

### 4.2. Study Design

Group sizes were determined on the basis of previous behavioral pharmacology studies performed in the Dom/Sub mouse lines and prior THC experiments in this model [31,52], in which comparable sample sizes were sufficient to detect robust treatment-by-phenotype effects. In accordance with the 3Rs principle, the number of animals was kept to the minimum expected to detect large behavioral effects while avoiding unnecessary animal use. Depending on the behavioral assay and availability of valid datasets after exclusion of technically invalid trials, final group sizes ranged from *n* = 7 to 10 animals per group.

**Figure 6:**
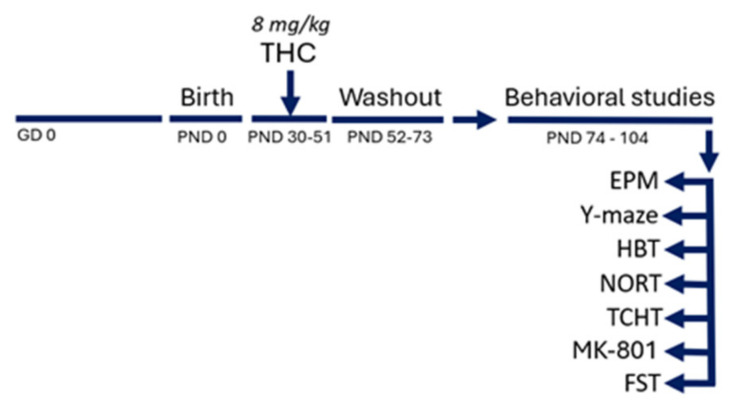
Study design for adolescent THC exposure and subsequent behavioral assessment.

The overall experimental timeline is shown in Figure 6. Offspring were born at PND 0. Δ^9^-tetrahydrocannabinol (THC) was administered at a dose of 8 mg/kg during adolescence, between PND 30 and 51. This was followed by a washout period from PND 52 to 73. Behavioral studies were conducted between PND 74 and 104 and included the elevated plus maze (EPM), Y-maze, hole-board test (HBT), novel object recognition test (NORT), three-chamber test (TCHT), MK-801-induced locomotor activity test, and forced swim test (FST). Abbreviations: GD—gestational day; PND—postnatal day; THC—Δ^9^-tetrahydrocannabinol; EPM—elevated plus maze; HBT—hole-board test; NORT—novel object recognition test; TCHT—three-chamber test; MK-801—dizocilpine, a non-competitive NMDA receptor antagonist; FST—forced swim test.

### 4.3. Drugs

#### 4.3.1. THC

The THC preparation involved dissolving 3.14 g of cannabidiol in 100 mL dry dichloromethane, dispersing 400 mg pre-heated MgSO_4_, and adding 25 µL of BF_3_ diethyl etherate. The reaction was mixed at −15 °C for 1.5 h in an N_2_ atmosphere, then quenched with 20 mL ice-cold NaHCO_3_ saturated solution. Phases were separated, and the aqueous phase was extracted with two portions of 30 mL dichloromethane (DCM). The combined organic phase was washed to neutral with saturated NaCl solution, dried over MgSO_4_, and evaporated. The yield of THC was 82.3%, with the rest remaining unreacted CBD. The purity of THC used for treatment was >97%, confirmed by LC-MS.

THC was resuspended in an 18:1:1 mixture of saline: ethanol: Tween^®^ 80 (Sigma-Aldrich, Rehovot, Israel, Cat. No. P1754) and administered at a dose of 8 mg/kg s.c. in 0.1 mL bolus for 21 days (every day, days 30–51). The dose and treatment regimen were chosen based on previous observations of THC effects on Dom and Sub mice (dose found effectively psychotropic in the previous studies, and thus suitable to parallel adolescent recreational THC exposure) [52,57].

#### 4.3.2. MK-801

MK-801 [(+)-5-methyl-10,11-dihydroxy-5H-dibenzo (a,d) cyclohepten-5,10-imine], also known as dizocilpine hydrogen maleate, is an effective anticonvulsant with both anxiolytic and sympathomimetic properties and is used as an antagonist of the N-methyl-D-aspartate glutamate receptor of the central nervous system (CNS). MK-801 (Sigma-Aldrich, Cat. No. 475878) was dissolved in normal saline (0.9% NaCl) and administered at a dose of 0.5 mg/kg i.p. in a 0.2 mL bolus, 1 h before behavioral assay.

### 4.4. Behavioral Tests

#### 4.4.1. Hole-Board Test (HBT)

The HBT is used for assessing exploratory behaviors in rodents [58,59]. The apparatus consists of a square-shaped, clear polycarbonate box (40 cm × 40 cm × 35 cm) with an inserted elevated metal floor arena (15 cm above box bottom) containing 16 equally spaced holes (3 cm diameter). Each mouse was placed in the center of the hole-board and allowed to explore for 5 min. The frequency of spontaneous elicited hole-poking behavior (number of head dips) was manually recorded, and all sessions were video recorded. Head-dipping behavior indicates exploratory activity, with decreased head-dipping suggesting low exploratory activity and increased head-dipping indicating high exploration.

#### 4.4.2. Novel Object Recognition Test (NORT)

NORT is a commonly used behavioral assay for investigating various aspects of learning and memory in mice [60,61]. A mouse is presented with two similar objects during a training session, and then one of the objects is replaced by a new object during a testing session. The time taken to explore the new object serves as an index of recognition memory. NORT was performed in open field square arenas. The test comprised four sessions: (1) habituation, (2) training, (3) test 1 (30 min retention interval), and (4) test 2 (24 h retention interval). Preference (%) for a novel object was calculated as (novel object exploration time (TN)/(TN + familiar object exploration time (TF))) × 100. The discrimination index (DI) was calculated as (TN − TF)/(TN + TF), ranging from +1 to −1, where positive values indicate preference for the novel object. Animal behavior was recorded by EthoVision 11.5 tracking software.

#### 4.4.3. Three-Chamber Test (TChT)

The TChT is used to assess social behavior in rodents. The device consists of three chambers, each 19 × 45 cm. In the first part, the test mouse was habituated to the testing environment for 5 min. In the second phase, a stranger mouse in a cylinder was added to a random room for 10 min. The number of entries into each side chamber was measured for 10 min. Mouse movements were recorded using EthoVision 11.5.

#### 4.4.4. MK-801-Induced Locomotor Activity Test

The effect of MK-801 on locomotor activity was assessed in the open field (OF) apparatus. The experimental design included three sessions: (1) habituation (30 min), (2) baseline activity measurement after saline injection (30 min), and (3) activity measurement upon administration of a single dose of MK-801 (180 min).

#### 4.4.5. Elevated Plus Maze (EPM)

EPM assesses anxiety-like and emotional behavior in rodents [62]. The apparatus (54 cm high, 66 cm long) has two open and two closed arms arranged in a “+” shape. Mice were placed in the center facing a closed arm, and behavior was recorded using EthoVision 11.5 for 5 min. Anxiety-like behavior (open/closed arm time and entry ratio), locomotor activity (distance, speed) and exploratory behavior (entries and time in each arm) were analyzed [62,63].

#### 4.4.6. Y-Maze Spontaneous Alternation (SA) Test

This test assesses short-term spatial working memory based on rodents’ natural tendency to explore novel environments [64]. The maze consists of three black opaque plastic arms (38 cm each) positioned at 120° angles. At the start of the test, a mouse is placed at the end of one arm and allowed to freely explore the maze for 5 min. The arms are labeled A, B, and C. An arm entry is counted when all four limbs enter an arm. An alternation is defined as consecutive entries into all three arms. The percentage of spontaneous alternation is calculated as: % Alternation = (Number of alternations/[Total arm entries − 2]) × 100. Higher alternation percentages indicate better working memory. Behavior is recorded and analyzed using EthoVision 11.5 (Noldus, Wageningen, The Netherlands).

#### 4.4.7. Forced Swim Test (FST)

FST is used to assess stress-coping strategies in rodents rather than depression-like behavior [65]. Each mouse is placed individually into a glass cylinder (30 cm height, 10 cm diameter) filled with water (25 ± 2 °C) to a depth of 25 cm and forced to swim for 6 min. Immobility, defined as floating with minimal movements to remain afloat. Mice that fail to stay afloat are removed immediately. Increased immobility reflects a passive stress-coping strategy, whereas reduced immobility reflects an active coping strategy [66].

### 4.5. Statistical Analysis

Statistical analysis was performed using GraphPad Prism software version 10.0. Statistical significance between animal groups was assessed using one- and two-way ANOVA followed by a Tukey or Sidak post hoc test, or a two-tailed Student’s *t*-test, depending on the experiment type. Graphs present data as mean ± SEM. Statistical differences were indicated as: * at *p* < 0.05, ** at *p* < 0.01, *** at *p* < 0.001, and **** at *p* < 0.0001.

## Data Availability

The raw data supporting the conclusions of this article will be made available by the authors on request.

## Supplementary Materials

The following supporting information can be downloaded at: https://www.mdpi.com/article/10.3390/ph19071009/s1.

## Abbreviations

The following abbreviations are used in this manuscript:

THC	Δ^9^-tetrahydrocannabinol
HPA	hypothalamic–pituitary–adrenal axis
NMDA	N-methyl-D-aspartate
MK-801	[(+)-5-methyl-10,11-dihydroxy-5H-dibenzo (a, d) cyclohepten-5,10-imine]
Dom	Dominant
Sub	Submissive
HBT	Hole-board test
NORT	Novel object recognition test
DI	discrimination index
TChT	three-chamber test
DCM	dichloromethane
CNS	central nervous system
OF	Open field
