# Supplementary material for: Adolescent Δ9-THC Exposure Differentially Affects Mice Depending on Their Personality
Source: Pharmaceuticals (Basel). 2026 Jun 29;19(7):1009. doi: 10.3390/ph19071009 (PMC13414570; doi:10.3390/ph19071009)
Supplement: Supplementary file 1 [file pharmaceuticals-19-01009-s001.zip › pharmaceuticals-4342613-supplementary.pdf]

# Supplementary Materials

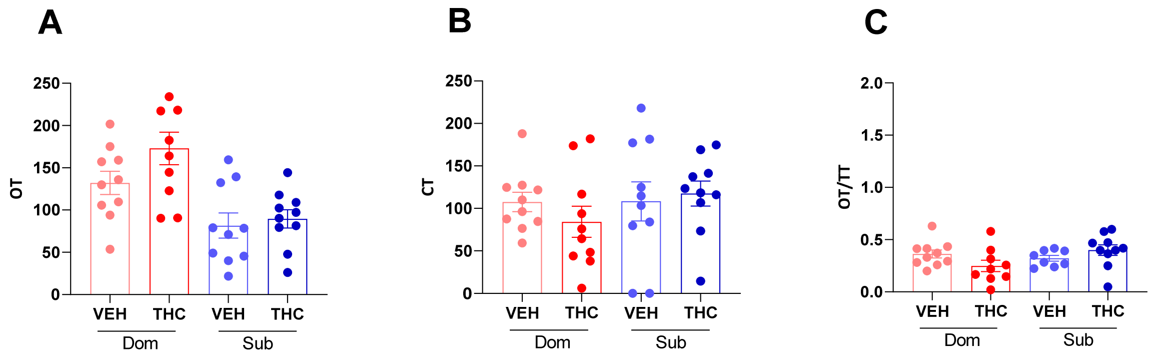


***Suppl. Figure S1.*** **Behavior in EPM test in Dom and Sub mice after adolescent exposure to Δ$^9$-THC.** Statistical significance between groups was assessed using two-way ANOWA with Sidak test. Error bars indicate SEM (n = 10/group). Abbreviations: OT-time spent in open arms; CT- time spent in close arms; TT- total time; OT/TT-anxiety level.

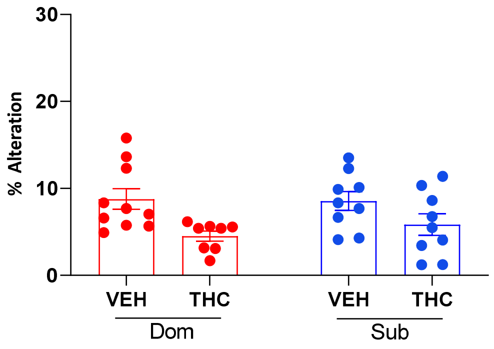


***Suppl. Figure S2.*** **Behavior in Y-maze spontaneous alternation in Dom and Sub mice after adolescent exposure to Δ$^9$-THC.** Differences between groups were assessed using a two-way ANOWA test. In figure shown % alterations in Y maze test. n=8-10. Error bars indicate SEM.

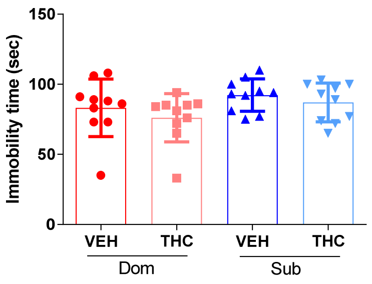


***Suppl. Figure S3.*** **Behavior in FST in Dom and Sub mice after adolescent exposure to Δ$^9$-THC.** Differences between groups were assessed using a two-way ANOWA test. In figure shown % alterations in Y maze test. n=8-10. Error bars indicate SEM.
